# Flourishing Japanese Encephalitis, Associated with Global Warming and Urbanisation in Asia, Demands Widespread Integrated Vaccination Programmes

**DOI:** 10.5334/aogh.2580

**Published:** 2019-07-30

**Authors:** Ryo Sakamoto, Tetsuya Tanimoto, Kenzo Takahashi, Tamae Hamaki, Eiji Kusumi, Andy Crump

**Affiliations:** 1 Medical Governance Research Institute, Tokyo, JP; 2 Navitas Clinic, Tokyo, JP; 3 Teikyo University Graduate School of Public Health, Tokyo, JP; 4 Kitasato Institute for Life Sciences, Kitasato University, Tokyo, JP

**Figure 1:**
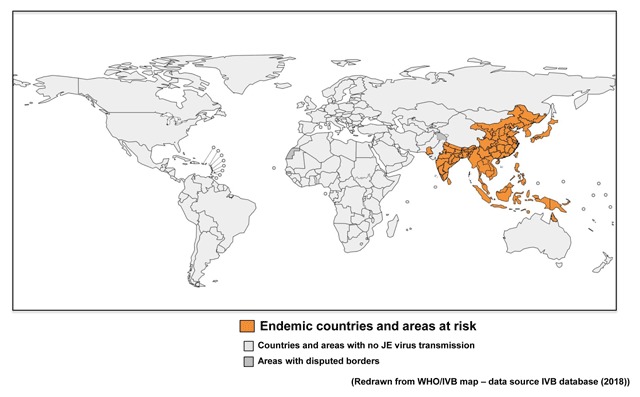
Japanese encephalitis: global distribution.

First described in Japan in 1871, Japanese encephalitis (JE) virus is the most important vaccine-preventable cause of encephalitis in Asia. Over 3 billion people live in countries with JE virus transmission risk areas, predominantly in rural locations where the virus exists in a cycle involving pigs, horses, wading birds, and mosquitoes, which also transmit it to humans (Figure 1). Some 75% of cases occur in young children, with adults having developed immunity, which weakens in old age [1]. There is no cure for the disease and JE cases are primarily asymptomatic. But 1 in 200 infections lead to severe disease, resulting in a fatality rate approaching 30%, with 30–50% of survivors suffering long-term neurological sequelae.

Vaccination is the mainstay of JE control and prevention, with surveillance and immunisation improving significantly throughout much of Asia over the past 20 years. JE immunisation represents a good return on investments, vaccination saving an estimated US $200 per care-seeking case and US $35,000 per death [2]. Ideally, appropriate, safer, cost-effective vaccines, such as China’s low-cost CD-JEV live-attenuated vaccine, supported by PATH and already administered to 400 million Asian children, should replace higher-cost inactivated ones. Nevertheless, reported cases of JE, which can occur in epidemics, have remained relatively constant, ostensibly due to a combination of the impact of global warming, increased flooding, and movement of pig and rice farming to peri-urban areas. Global warming can cause not only mosquito vectors to invade higher elevations and latitudes but also an increase in rainfall, facilitating increased breeding of JE vectors. In addition, in warmer climates, female mosquitoes take a bloodmeal more frequently, enhancing the likelihood of viral transfer.

In Japan, JE vaccination was historically not required for individuals living on the northern Hokkaido island. However, a recent northward spread of *Culex tritaeniorhynchus*, the mosquito vector that transmits JE virus to humans, resulted in commencement of a JE vaccination programme in 2016 [3]. In the Himalayas, where the JE virus was commonly detected at 1000–2000 metres during 2004–2005, it was found at over 3000 metres during 2006–2008 [4]. In India’s north-eastern hilly, flood-prone Assam State, annual JE cases rose almost five-fold between 2010–2014, causing annual deaths to rise from 41 to 160 [5]. In Nepal, cases increased twelve-fold between 2011–2015, leading to the expansion of the JE vaccination programme nationwide during 2015–2016 [1]. Furthermore, JE vectors are salt-tolerant, and mosquito numbers may increase with rising sea levels. In Indonesia, inundation has occurred annually from 2013, with Bali reporting 408 JE cases during 2014–2016 [6].

Within Asia, JE problems have increased, driven by a population that has more than doubled in the last 50 years, accompanied by a concomitant increase in demand for rice and meat. JE traditionally occurs in rural areas where pig and rice farming is commonplace. Rapid economic growth and urbanisation have increased demands for food, bringing pig and rice farming into urban and peri-urban areas. Pan-Asian meat production rose almost 800% during 1967–2015, and by 17% over the last decade, resulting in a massive increase in virus-amplifying hosts [7]. Vector Culex mosquitoes commonly breed in rice paddies and over the past four decades, the area under rice cultivation in all JE-endemic countries (excluding the Russian Federation and Australia) has increased by 22%, to 1,234,000 km^2^. Overall, the rural population living in areas at risk for JE has increased by 66% in the last 40 years [8].

In Viet Nam’s Can Tho city, JE virus-positive mosquitoes proliferated, with the presence and number of pigs positively correlating with increased numbers of vector mosquitoes [9,10]. During 2011–2015, JE cases in Viet Nam doubled, confirming that JE now exists in rural areas while also posing health risks to urban dwellers, travellers, and tourists visiting all such locations for sightseeing and business [11].

**Figure 2:**
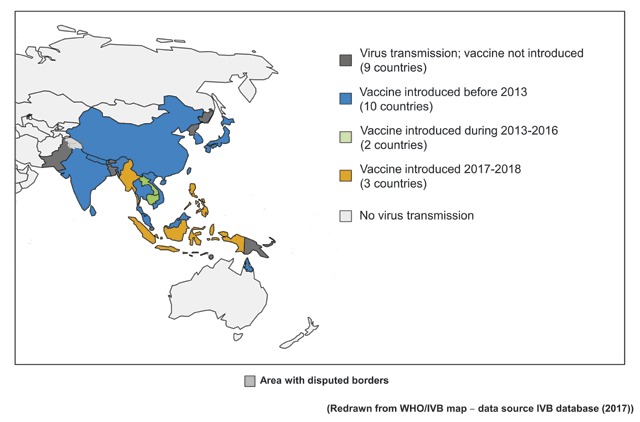
Japanese encephalitis: regional immunisation status.

**Figure 3:**
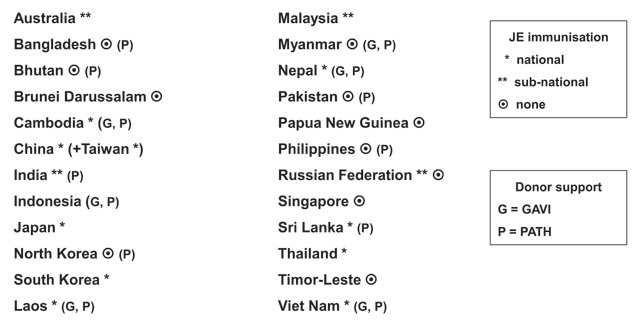
Japanese encephalitis: affected countries & vaccination programmes (2016).

Recently, positive steps have been taken to tackle JE. In 2016, 22 of the 24 countries with JE virus transmission risks conducted routine disease surveillance, with laboratory diagnostic facilities in many becoming more widely available. Wealthier countries in the region, notably Japan, China, and South Korea, have operated successful JE immunisation projects for decades, but in resource-poor Asian nations, vaccination has been patchy (Figure 2). Through a generous, one-off and time-limited (2003–2009) grant from the Bill & Melinda Gates Foundation, the PATH organisation initiated a JE project with the goal of eliminating clinical JE and avoiding the unnecessary death and disability caused by the disease. PATH has already introduced and expanded immunisation programmes in 13 countries in the region. In addition, the Global Alliance for Vaccines and Immunisation (GAVI) began funding JE immunisation programmes in 2015, allocating $52 million to be spent during 2016–2020, enough to immunise an estimated 70 million people and prevent 8,000 deaths. However, GAVI financial support is only provided to “GAVI-eligible” countries, with only 6 of the 14 “eligible” receiving funds by 2018 (Figure 3).

Successful ongoing vaccination interventions have radically reduced JE incidence in resource-rich countries, while several “donor-selected” nations have now switched to more advantageous, less reactogenic vaccines (such as CD-JEV) administered via simpler dosing schedules. Presently, political constraints, public mistrust, and lack of a regionally coordinated response, not scientific, medical, or financial factors, may be the reasons that the world is still facing a JE threat. Lack of access to available funds and the most cost-effective vaccine, anti-vaxx sentiments, increasing public apathy towards childhood immunisation, together with a failure to develop a cohesive regional immunisation plan of action, means that millions of people, mostly young children, remain at risk throughout much of Asia. A fragmented, poorly-coordinated regional response is unlikely to accomplish the desired JE disease control. Moreover, since the 1990s, of the five JE viral strains, the GIII strain has been the most widely distributed genotype, with most licensed JE vaccines being derived only from the GIII strain. A recent major shift in the dominant viral genotype circulating in JE-endemic areas, with G1 replacing GIII in many countries means that if the strains of different genotypes exhibit antigenic variations, vaccine efficacy may be compromised [12].

Despite the recent progress, it is clear that a well-orchestrated regional vaccination initiative, covering all JE-endemic countries, should be extensively and intensively promoted [13]. We call for concerted international action resulting in the introduction and dissemination of nationwide, integrated routine JE immunisation programmes to protect all citizens, particularly children, especially those in hitherto non-exposed populations, as well as the burgeoning number of travellers flocking to various parts of Asia.
